# Gait Initiation in Parkinson’s Disease: Impact of Dopamine Depletion and Initial Stance Condition

**DOI:** 10.3389/fbioe.2020.00137

**Published:** 2020-03-06

**Authors:** Chiara Palmisano, Gregor Brandt, Matteo Vissani, Nicoló G. Pozzi, Andrea Canessa, Joachim Brumberg, Giorgio Marotta, Jens Volkmann, Alberto Mazzoni, Gianni Pezzoli, Carlo A. Frigo, Ioannis U. Isaias

**Affiliations:** ^1^Department of Neurology, University Hospital Würzburg and The Julius Maximilian University of Würzburg, Würzburg, Germany; ^2^MBMC Lab, Department of Electronics, Information and Bioengineering, Politecnico di Milano, Milan, Italy; ^3^Translational Neural Engineering Area, The Biorobotics Institute, Scuola Superiore Sant’Anna, Pontedera, Italy; ^4^Fondazione Europea di Ricerca Biomedica (FERB Onlus), Cernusco s/N (Milan), Italy; ^5^Department of Nuclear Medicine, University Hospital Würzburg and The Julius Maximilian University of Würzburg, Würzburg, Germany; ^6^Department of Nuclear Medicine, Fondazione IRCCS Ca’ Granda – Ospedale Maggiore Policlinico, Milan, Italy; ^7^Centro Parkinson, ASST G. Pini-CTO, Milan, Italy

**Keywords:** gait initiation, Parkinson’s disease, basal ganglia, dopamine, base of support, anthropometric measurements

## Abstract

Postural instability, in particular at gait initiation (GI), and resulting falls are a major determinant of poor quality of life in subjects with Parkinson’s disease (PD). Still, the contribution of the basal ganglia and dopamine on the feedforward postural control associated with this motor task is poorly known. In addition, the influence of anthropometric measures (AM) and initial stance condition on GI has never been consistently assessed. The biomechanical resultants of anticipatory postural adjustments contributing to GI [imbalance (IMB), unloading (UNL), and stepping phase) were studied in 26 unmedicated subjects with idiopathic PD and in 27 healthy subjects. A subset of 13 patients was analyzed under standardized medication conditions and the striatal dopaminergic innervation was studied in 22 patients using FP-CIT and SPECT. People with PD showed a significant reduction in center of pressure (CoP) displacement and velocity during the IMB phase, reduced first step length and velocity, and decreased velocity and acceleration of the center of mass (CoM) at toe off of the stance foot. All these measurements correlated with the dopaminergic innervation of the putamen and substantially improved with levodopa. These results were not influenced by anthropometric parameters or by the initial stance condition. In contrast, most of the measurements of the UNL phase were influenced by the foot placement and did not correlate with putaminal dopaminergic innervation. Our results suggest a significant role of dopamine and the putamen particularly in the elaboration of the IMB phase of anticipatory postural adjustments and in the execution of the first step. The basal ganglia circuitry may contribute to defining the optimal referent body configuration for a proper initiation of gait and possibly gait adaptation to the environment.

## Introduction

In the case of Parkinson’s disease (PD), locomotion is one of the functionally relevant daily acts that can be severely affected, especially at the initiation of movement. Indeed, gait initiation (GI) greatly challenges the balance control system as the subject moves from a stable balance condition to unstable single limb support during locomotion. This motor task can be specifically impaired in parkinsonian patients due to start hesitation or gait freezing, leading to falls, injuries, fear of falling, and restriction of activities (Peterson and Horak, 2016). The identification of a behavioral measurement that can reliably describe balance disturbances in PD is urgently needed.

Gait initiation includes the production of anticipatory postural adjustments (APA), a centrally mediated feedforward motor program (Massion, 1992; MacKinnon et al., 2007) aimed in this context at destabilizing the antigravity postural set for the subsequent execution of a functionally optimized step (Crenna and Frigo, 1991). In the mechanics of GI, APA manifest as an initial displacement of the center of pressure (CoP) in the posterior direction and toward the swing foot (i.e., the one that will move first), thus creating an offset between center of mass (CoM) and CoP and a consequent gravitational momentum favoring the forward acceleration of CoM and its positioning over the stance foot (Crenna and Frigo, 1991). More specifically, two phases can be identified in preparation of GI, the imbalance (IMB) and the unloading (UNL) phase. The IMB phase corresponds to the first CoP displacement backwards and toward the swing foot. The UNL phase is the subsequent displacement of the CoP toward the stance foot, prominently in the mediolateral (ML) direction, needed to transfer the load onto the supporting foot and to allow the swing foot to clear the ground and make a step (Crenna and Frigo, 1991; Crenna et al., 2006).

The contribution of the basal ganglia and dopamine on postural control is still largely unknown, but indirect evidence points toward their possible involvement in the production of APA. Firstly, the striatum is involved in the feedforward motor control (Massion, 1992) and striatal dopamine deficiency is known to primarily affect learning and consolidation processes of motor programs (Marinelli et al., 2009; Isaias et al., 2011) and thus possibly the APA. Secondly, we expect a reduced energetic cost associated with a correct execution of APA (Anand et al., 2017) and movement-related energetic tradeoff is directly modulated by the striatal dopaminergic activity (Mazzoni et al., 2007).

Clinical studies on GI in subjects with PD, a predominant dopamine deficiency syndrome (Isaias et al., 2006), showed, however, conflicting results: some described hypometric and prolonged APA during GI compared to healthy subjects (Burleigh-Jacobs et al., 1997; Halliday et al., 1998; Crenna et al., 2006), while others did not show any pathological difference (Schlenstedt et al., 2018). We hypothesize that the poor agreement in the literature might be directly related to the different experimental conditions adopted to investigate GI in parkinsonian patients. Firstly, little or no attention has been given to the influence of the anthropometric measures (AM) and of the base of support (BoS), although these parameters could significantly impact this motor task (Rocchi et al., 2006). Moreover, the BoS is directly influenced by postural instability, which is a cardinal motor feature of PD (Peterson and Horak, 2016). In this regard, imposing a standardization of the BoS [e.g., a predefined distance between the feet (Halliday et al., 1998; Crenna et al., 2006; Jacobs et al., 2009; Mouchnino et al., 2015)] might be critical since it can alter the subjects’ natural motor behavior. Secondly, the contribution of the dopaminergic circuitry in APA production has been exclusively investigated by describing the acute effect of levodopa assumption. However, a dopaminergic replacement therapy does not necessarily restore the biomechanical properties of GI (Curtze et al., 2015), nor does it affect only the dopaminergic striatal processing, but it can influence other brain areas, such as the supplementary motor area (SMA) (Turco et al., 2018), which is known to be involved in the generation of APA at GI (MacKinnon et al., 2007; Jacobs et al., 2009; Mouchnino et al., 2015).

The aim of this work was to define which biomechanical resultants of APA at GI are PD-related and dopamine (putamen)-dependent, accounting for the influence of AM and the BoS.

## Materials and Methods

### Study Subjects

We recruited 26 subjects with a clinical diagnosis of idiopathic PD and 27 age-matched healthy controls (HC). PD was diagnosed according to the United Kingdom Brain Bank Clinical Diagnostic criteria. We included only subjects capable of completing at least three GI trials without assistance (range: 3–6). Exclusion criteria were neurological diseases other than PD, cognitive decline (Mini-Mental State Examination score ≥ 27), vestibular disorders, cardiovascular diseases (including symptomatic postural hypotension), diabetes, orthopedic problems, or past major orthopedic surgery. We also excluded patients suffering from start hesitation, freezing of gait and levodopa-related motor fluctuations (e.g., dyskinesia). A neurologist expert in movement disorders (IUI) clinically evaluated all patients using the Unified Parkinson’s Disease Rating Scale motor part (UPDRS-III). Demographic and clinical data are shown in Table 1. The local ethical committee approved the study and all subjects gave written informed consent according to the Declaration of Helsinki.

**TABLE 1:** List of the biomechanical parameters analyzed.

Acronym	Description	Decomposition
**Anthropometric measurements (AM)**		
BH	Body height (cm)	
IAD	Inter anterior superior iliac spine distance (cm)	
LL	Limb length (cm)	
FL	Foot length (cm)	
BM	Body mass (kg)	
BMI	Body mass index (kg/cm^2^)	
**Base of support (BoS)**		
BA	Base of support area (cm^2^)	
BoSW	Base of support width (cm)	
FA	Foot alignment (cm)	
β_Δ_	Difference between feet extra-rotation angles (°)	
β (°)	BoS opening angle (°)	
**Imbalance phase**		
IMBT	Imbalance duration (s)	
IMBD	Imbalance CoP displacement (mm)	AP, ML
IMBAV	Imbalance CoP average velocity (mm/s)	AP, ML
IMBMV	Imbalance CoP maximal velocity (mm/s)	AP, ML
IMBCoMV	CoM velocity at imbalance end (m/s)	
IMBCoMA	CoM acceleration at imbalance end (m/s^2^)	
IMBCoPCoM	CoP-CoM distance at imbalance end (m)	
IMBSLOPE	Orientation of CoP-CoM vector with respect to the progression line at imbalance end (deg)	
HOCoPD	CoP distance from the line passing through the markers on the heels at swing heel off (%FL)	AP
**Unloading phase**		
UNLT	Unloading duration (s)	
UNLD	Unloading CoP displacement (mm)	AP, ML
UNLAV	Unloading CoP average velocity (mm/s)	AP, ML
UNLMV	Unloading CoP maximal velocity (mm/s)	AP, ML
UNLCoMV	CoM velocity at unloading end (m/s)	
UNLCoMA	CoM acceleration at unloading end (m/s^2^)	
UNLCoPCoM	CoP-CoM distance at unloading end (m)	
UNLSLOPE	Slope of CoP-CoM vector at unloading end (deg)	
TOCoPD	CoP distance from the line passing through the markers on the heels at the swing foot toe off (% FL)	AP
**Stepping phase**		
TOCoMV	CoM velocity at stance foot toe off (m/s)	
TOCoMA	CoM acceleration at stance foot toe off (m/s^2^)	
TOCoPCoM	CoP-CoM distance from the line passing through the markers on the heels at the stance foot toe off (m)	
SL	First step length (m)	
SAV	First step average velocity (m/s)	
SMV	First step maximal velocity (m/s)	

*The imbalance phase mediolateral CoP displacement (IMBD ML) was considered positive when the shift of the CoP was toward the swing foot, while the unloading phase mediolateral CoP displacement (UNLD ML) was considered positive when the CoP was moving toward the stance foot. The imbalance phase anteroposterior CoP displacement (IMBD AP) and unloading phase anteroposterior CoP displacement (UNLD AP) were both defined positive when the CoP movement was oriented backward. AP, anteroposterior; ML, mediolateral.*

### Experimental Setup

Patients were evaluated in the morning after overnight suspension of all dopaminergic drugs (meds-off, PD-off). A subset of 13 patients (PD”-off) also executed the task 1 h after the oral intake of 200/50 mg fast-release soluble levodopa/benserazide (meds-on, PD”-on). We recorded motor performance with an optoelectronic system (six cameras SMART-DX, BTS) and two dynamometric force plates (9260aa, KISTLER).

**FIGURE 1:**
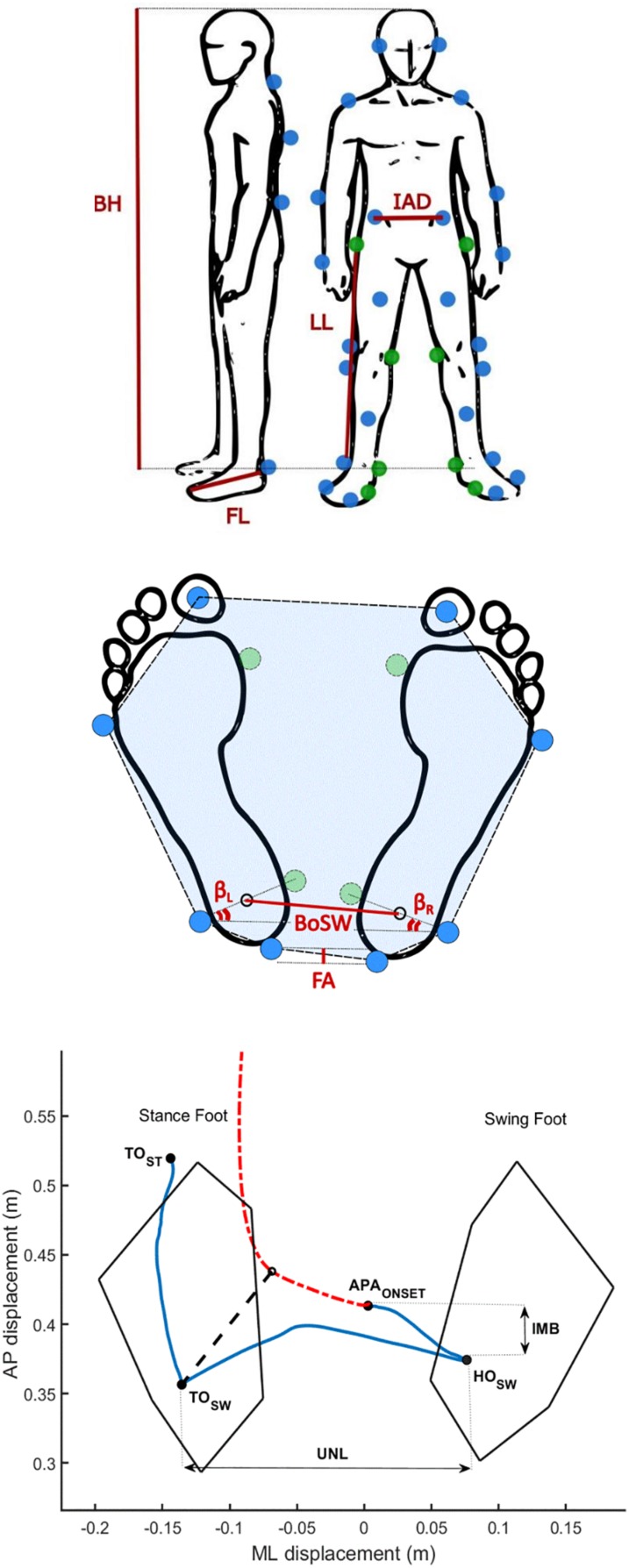
Biomechanical measurements. **(A)** Marker protocol and anthropometric measurements. Blue dots show the position of the markers applied for the gait initiation (GI) trials. Green dots show the positions of the eight additional markers used only for the calibration trial to define the main anthropometric measurements (AM): body height (BH), inter anterior-superior iliac spine distance (IAD), limb length (LL), and foot length (FL). Body mass (BM) and body mass index (BMI) were calculated by means of recordings with force plates. The intermetatarsal and intermalleolar distance of each foot were also computed. **(B)** Base of support (BoS) parameters. The BoS area (BA) is the area inside the dashed line (highlighted in blue). The BoS width (BoSW) was computed as the distance between the ankle centers. We additionally defined the foot alignment (FA), the left (βL), and the right (βR) feet extra-rotation angles, and their difference (βΔ = | βL-βR|). The sum of βL and βR defined the BoS opening angle (β). **(C)** Example of the pathway of the center of pressure (CoP, blue solid line) and center of mass (CoM, red dash-dotted line) during a GI trial of one healthy subject. The imbalance (IMB) and unloading (UNL) phases were analyzed based on the path traveled by the CoP before the completion of the first step. We defined the IMB phase as the interval between the onset of the APA (APA_ONSET_) and the heel off of the swing foot (HO_SW_), and the unloading phase (UNL) as the interval between the HO_SW_ and the toe off of the swing foot (TO_SW_). The black dashed line represents the CoP-CoM vector at the end of the UNL phase. TO_ST_ is the instant of the toe off of the stance foot.

Subjects were instructed to stand quietly on the force plates (one foot on each) for about 30 s. Following a verbal cue, subjects waited for a self-selected time interval before walking to the end of the walkway, leading with their self-selected stepping leg, and moving at their own (spontaneous) pace. The feet position during initial standing was self-selected by each subject. Kinematics was monitored with a full body marker set of 29 markers placed on anatomical landmarks (Figure 1A) according to a published protocol (Palmisano et al., 2019).

### Biomechanical Evaluation

#### Anthropometric Measurements and Base of Support

The AM were computed over a period of 5 s of standing upright on the force plates using eight additional markers placed on both sides of the body on the following landmarks: the first metatarsal head, the medial malleolus, the medial femoral condyle and greater trochanter (calibration trial, Figure 1A; Palmisano et al., 2019). For each subject, we evaluated the following AM: body height (BH), inter anterior-superior iliac spine distance (IAD), limb length (LL), foot length (FL), body mass (BM), and body mass index (BMI). The intermetatarsal and intermalleolar distance of each foot were also computed and served to estimate the position of the first metatarsal heads and the medial malleoli during the GI trials. The BoS was defined for each GI trial by the markers placed on the feet and by the AM (Figure 1B). In particular, we computed the BoS area (BA) as the area of the polygon described by the markers placed on the heels, the lateral malleoli, the fifth metatarsal bones and the hallux. The BoS width (BoSW) was calculated as the distance between the ankle joint centers, estimated as the mid points between the lateral and medial malleoli. To account for possible asymmetry in the feet placement, we also defined: (i) the foot alignment (FA), as the anteroposterior (AP) distance between the two markers placed on the heels, (ii) the difference (βΔ) between the left (βL) and the right (βR) feet extra-rotation angles, i.e., the angle between the axis passing through the lateral and medial malleoli and the horizontal axis of the reference system of the laboratory, and (iii) the BoS opening angle (β), estimated as the sum of βL and βR. Abbreviations of the AM and BoS parameters are listed in Table 1. The AM and BoS values are described in Table 2.

**TABLE 2:** Demographic, clinical, and biomechanical data.

		HC	PD-off	PD”-off	PD”-on
DEM	Gender (males/total)	17/27(∼63%)	18/26(∼69%)	8/13(∼61%)	8/13(∼61%)
	Age (years)	61.22 (5.15)	61.03 (7.94)	61.62 (9.13)	61.62 (9.13)
AM	BH (cm)	170.7 (9.8)	171.1 (10.8)	170.1 (11.5)	170.1 (11.5)
	LL (cm)	89.1 (4.8)	88.7 (6.9)	89.3 (8.4)	89.3 (8.4)
	FL (cm)	25.1 (1.6)	25.2 (1.6)	25.2 (1.9)	25.2 (1.9)
	BM (Kg)	75.25 (12.66)	75.57 (16.71)	69.93 (13.15)	69.93 (13.15)
	BMI (kg/cm^2^)	25.51 (3.56)	25.63 (4.31)	24.07 (3.40)	24.07 (3.40)
	IAD (cm)	28.2 (2.9)	27.1 (2.6)	26.3 (2.2)	26.3 (2.2)
BoS	BA (cm^2^)	713.21 (105.27)	672.89 (108.07)	667.56 (84.33)	680.27 (129.79)
	BoSW (cm)	18.14 (3.97)	16.46 (3.59)	16.72 (3.21)	17.68 (3.50)
	FA (cm)	0.68 (0.36)	0.79 (0.53)	0.89 (0.46)	0.94 (0.34)
	β_Δ_ (°)	6.94 (4.86)	5.34 (3.77)	5.89 (3.89)	5.41 (3.69)
	β (°)	40.20 (14.58)	40.73 (11.45)	36.83 (11.67)	36.07 (13.90)
Clinical data	Disease duration (years)	–	10.85 (5.06)	10.84 (4.51)	10.84 (4.51)
	Hoen & Yahr (I–V stage)	–	2.62 (0.50)	2.62 (0.51)	2.62 (0.51)
	UPDRS-III (0–108 score)	–	28.87 (9.74)	26.36 (7.63)	9.54 (4.78)
	LEDD (mg)	–	893.04 (514.47)	985.25 (637.62)	985.25 (637.62)
Biomechanical	IMBT (s)	0.40 (0.09)	0.41 (0.13)	0.41 (0.09)	0.42 (0.09)
parameters	IMBD (mm)	62.5 (20.3)	45.8 (22.3)	38.2 (19.4)	49.5 (19.5)
	IMBD ML (mm)	44.2 (15.0)	32.0 (16.3)	27.6 (14.4)	34.4 (15.8)
	IMBD AP (mm)	36.9 (15.1)	27.5 (16.1)	21.1 (13.7)	31.8 (13.4)
	IMBAV (mm/s)	175.3 (75.5)	130.6 (73.4)	103.5 (58.5)	129.4 (75.5)
	IMBAV ML (mm/s)	125.8 (57.6)	91.5 (53.1)	75.28 (44.2)	91.5 (56.0)
	IMBAV AP (mm/s)	103.6 (50.5)	78.2 (49.4)	57.0 (38.5)	82.4 (51.3)
	IMBMV (mm/s)	346.5 (145.0)	260.3 (156.7)	199.9 (127.9)	266.6 (135.1)
	IMBMV ML (mm/s)	265.3 (111.9)	207.1 (136.3)	163.7 (117.1)	203.1 (117.4)
	IMBMV AP (mm/s)	233.0 (107.3)	174.2 (100.3)	131.3 (73.25)	179.6 (91.1)
	HOCoPD (%FL)	30.48 (6.36)	32.21 (9.27)	35.11 (9.08)	37.51 (8.38)
	UNLT (s)	0.37 (0.08)	0.39 (0.10)	0.41 (0.08)	0.36 (0.09)
	UNLD AP (mm)	−13.4(18.3)	−1.76(16.4)	−2.22(19.95)	6.5 (22.1)
	UNLAV AP (mm/s)	64.3 (35.7)	39.7 (27.2)	44.8 (28.8)	54.7 (40.2)
	UNLMV AP (mm/s)	347.5 (146.5)	311.1 (136.3)	306.6 (133.8)	348.6 (152.3)
	UNLCoMV (m/s)	0.21 (0.06)	0.18 (0.05)	0.17 (0.06)	0.18 (0.07)
	UNLCoMA (m/s^2^)	1.37 (0.40)	1.40 (0.45)	1.35 (0.48)	1.71 (0.70)
	UNLCoPCoM (m)	0.08 (0.03)	0.08 (0.02)	0.08 (0.02)	0.09 (0.02)
	TOCoPD (%FL)	36.05 (7.96)	33.04 (9.31)	36.25 (7.06)	34.89 (5.43)
	TOCoMV (m/s)	0.86 (0.13)	0.74 (0.19)	0.67 (0.19)	0.83 (0.18)
	TOCoMA (m/s^2^)	1.83 (0.51)	1.42 (0.39)	1.40 (0.42)	1.93 (0.46)
	TOCoPCoM (m)	0.51 (0.32)	0.63 (0.25)	0.73 (0.14)	0.76 (0.13)
	SL (m)	0.60 (0.21)	0.46 (0.11)	0.43 (0.11)	0.50 (0.12)
	SAV (m/s)	0.99 (0.22)	0.85 (0.23)	0.80 (0.21)	0.96 (0.24)

*Only the biomechanical parameters not correlated with the base of support (BoS) are included in the table. Data are shown as mean (standard deviation). DEM, demographic features; AM, anthropometric measurements; BoS, base of support; LEDD, levodopa equivalent daily dose; all other abbreviations are described in Table 1.*

#### Gait Initiation Parameters

The APA at GI were defined and evaluated based on the CoP pathway recorded by means of the dynamometric force plates. Subjects stood on two force plates, one foot on each. Each force plate calculated the location of the CoP under the foot in contact with the platform. When both feet are in contact with the ground, the net CoP is located between the two feet, depending on the relative weight supported by each limb (Winter, 1995). According to the principle of static equilibrium, we computed the CoP position as the weighted mean of the signals recorded by the two force plates (Winter, 1995):

$$C\,o\,P=\frac{C\,o\,P_{L}\times R\,V_{L}+C\,o\,P_{R}\times R\,V_{R}}{R\,V_{L}+R\,V_{R}}$$

where CoP_L_ and CoP_R_ are the centers of pressure recorded by the force plates under the left and right foot, respectively, and RV_L_ and RV_R_ are the left and right vertical ground reaction forces. The resultant kinematic data were filtered with a 5th-order lowpass Butterworth filter [cut off frequency: 30 Hz (Muniz et al., 2012)]. Based on the CoP displacement, four timed events were automatically identified by *ad hoc* algorithms and checked by visual inspection through interactive software: (i) the onset of the APA (APA_ONSET_), (ii) the heel off of the swing foot (HO_SW_), (iii) the toe off of the swing foot (TO_SW_), and (iv) the toe off of the stance foot (TO_ST_) (Figure 1C). APA_ONSET_ was computed as the first frame in which the CoP shifts consistently backwards and toward the swing foot (Martin et al., 2002; Isaias et al., 2014). We defined HO_SW_ as the most lateral motion of the CoP toward the swing foot, while TO_SW_ as the time when CoP shifts from lateral to anterior motion (Isaias et al., 2014). The TO_ST_ was identified as the last frame recorded by the force plates (Martin et al., 2002; Crenna et al., 2006; Isaias et al., 2014). We defined the imbalance phase (IMB) as the interval between the APA_ONSET_ and the HO_SW_, and the unloading phase (UNL) as the interval between the HO_SW_ and the TO_SW_ (Crenna et al., 2006; Isaias et al., 2014; Figure 1C). IMB and UNL phases were characterized in the AP and ML directions in terms of duration (IMBT and UNLT), CoP displacement (IMBD and UNLD), average velocity (IMBAV and UNLAV), and maximum velocity (IMBMV and UNLMV). Please refer to Table 1 for a detailed list of the extracted parameters and abbreviations. We also computed the AP position of CoP from the line connecting the markers on the two heels at HO_SW_ (HOCoPD) and at TO_SW_ (TOCoPD), to account for subjects’ posture during the task. Kinematic measurements served to characterize the first step and the movement of the CoM. Marker traces were filtered with a 5th-order lowpass Butterworth filter [cut off frequency: 10 Hz (Palmisano et al., 2019)]. As for the stepping phase (from HO_SW_ to the subsequent heel strike of the same foot) the parameters extracted were the step length (SL) and the average and maximum velocity (SAV and SMV), based on the marker placed on the heel of the swing foot. The CoM was computed as previously described by Dipaola et al. (2016). In brief, the CoM was estimated as the weighted mean of the CoM of each body segment (CoMj):

$$Y_{C\,o\,M}=\sum_{j}\frac{Y_{j}\,m_{j}}{M}$$

where Y_CoM_ is the generic coordinate of the CoM, Y_j_ is the coordinate of the CoM of the j-th anatomical segment, m_j_ is the mass of the j-th body segment and M is the mass of the whole body. The position of the CoM of each anatomical segment CoMj as well as its mass were calculated according to the anthropometric tables and regression equations proposed by Zatsiorsky (1998). We calculated velocity and acceleration of the CoM and its position with respect to the CoP at the end of the IMB phase [HO_SW_] (IMBCoMV, IMBCoMA, and IMBCoPCoM, respectively), at the end of the UNL phase [TO_SW_] (UNLCoMV, UNLCoMA, and UNLCoPCoM, respectively) and at TO_ST_ (TOCoMV, TOCoMA, and TOCoPCoM, respectively). Lastly, we computed the orientation of the vector joining CoP and CoM at the end of the IMB phase [HO_SW_] and at the end of UNL phase [TO_SW_] (IMBSLOPE and UNLSLOPE, respectively) as a measure of the direction of CoM acceleration at the end of the two APA phases.

#### Variables Selection and Decorrelation Procedure

To investigate and disentangle the influence of the BoS and AM on the GI biomechanical parameters, we performed a partial correlation analysis, a technique that allows us to verify if a linear relationship exists between two variables whilst controlling for the effect of other parameters. We applied this analysis on the data of the PD-off and HC groups between: (i) the GI parameters and the BoS, controlling for the AM, and (ii) the GI parameters and the AM, controlling for the BoS. The GI parameters that correlated with the BoS in one or both groups were excluded from further analyses. We decided to adopt this conservative approach because the (normal) preferred BoS of each patient in the absence of PD is indeterminable. The GI parameters that significantly correlated with the AM in one or both groups were instead decorrelated as described by O’Malley (1996). In brief, for each cohort we defined a linear model of the relationship between the correlated AM and GI parameters. We then computed for each observation i-th the perpendicular distance (di) between the data point and the fitted line as:

$$d_{i}=\frac{y_{i}-m\,x_{i}-c}{\sqrt{m^{2}+1}}$$

where y_i_ and x_i_ are the GI parameter and the AM values of the i-th observation, respectively, and m and c are the angular coefficient and the constant of the linear model (y = mx + c) fitting the data. By definition, this distance is uncorrelated with the original AM and GI parameters used to build the model. The resulting decorrelated data were then used for comparisons between groups and for the correlation with the imaging data.

#### Molecular Imaging Evaluation

A subset of 22 patients performed a single-photon computed tomography (SPECT) with [^123^I]N-ω-fluoropropyl-2β-carbomethoxy-3β-(4-iodophenyl)tropane (FP-CIT) during their clinical workup. This radioligand binds selectively to the presynaptic dopamine reuptake transporters (DAT) and provides a reliable measurement of the brain dopaminergic innervation of the striatum (Isaias et al., 2006). The imaging data were processed with the Basal Ganglia Matching Tool (Nobili et al., 2013). We then investigated the correlation between the biomechanical values and the DAT density of the putamen, as the main motor structure in the striatum, contralateral to the swing foot (putamen_SWING_), and contralateral to the stance foot (putamen_STANCE_).

#### Statistical Analysis

For each patient, measurements were averaged over GI trials performed with the same swing foot. The influence of the BoS and AM on the GI parameters was investigated with a partial correlation analysis with the significance level set at ρ > 0.5 (Spearman’s ρ correlation coefficient) and *p* < 0.01. GI parameters (independent from BoS and decorrelated from AM) were compared between PD-off and HC with a Wilcoxon test. The effect of levodopa was investigated with a Wilcoxon matched pairs test [a pair being the same subject in meds-off (PD”-off) and meds-on condition (PD”-on)]. We then calculated the Spearman’s ρ correlation coefficient for GI parameters and DAT density of the putamen. The analyses for this study were performed with Matlab^®^ R2018b ambient (The MathWorks Inc., Natick, MA, United States) and JMP 14.0.0 (SAS Institute Inc., Cary, NC, United States).

## Results

### Study Subjects

Demographic features, AM and the initial stance condition (i.e., BoS) did not significantly differ between HC and PD-off and between PD”-off and PD”-on (Wilcoxon test and Wilcoxon matched pairs test, respectively, *p* < 0.05). All patients of the PD”-off group derived significant benefit with levodopa (range 23–82% improvement at UPDRS-III score, Wilcoxon matched pairs test *p* < 0.01) (Table 2).

### Influence of the Base of Support on Gait Initiation

In Table 2 we listed the biomechanical parameters that were not influenced by the BoS. Apart from the measures regarding the CoM, all other IMB features were observed to be independent from the BoS. In contrast most of the UNL parameters were influenced by the BoS and were thus excluded from further analyses. Therefore, analyses on the UNL phase cannot be conclusive.

### Parkinson’s Disease Effect on Gait Initiation

The disease *per se* mostly influenced the IMB phase and the stepping phase, whereas the UNL phase was less affected and showed a major involvement of the AP measurements (Tables 2, 3). Indeed, the IMB displacement (IMBD, IMBD ML, and IMBD AP) and the IMB average and maximum velocity (IMBAV, IMBAV ML, IMBMV, and IMBMV ML) were lower in the PD-off group than HC. Between these two cohorts, the UNL phase differed with regards to the AP displacement (UNLD AP) and average velocity (UNLAV AP). The CoM velocity and acceleration at the TO_ST_ (TOCoMV and TOCoMA), the first step length and the average velocity (SL and SAV) were reduced in the PD-off cohort with respect to HC.

**TABLE 3:** Statistical results of the comparisons between groups and their correlations with molecular imaging findings.

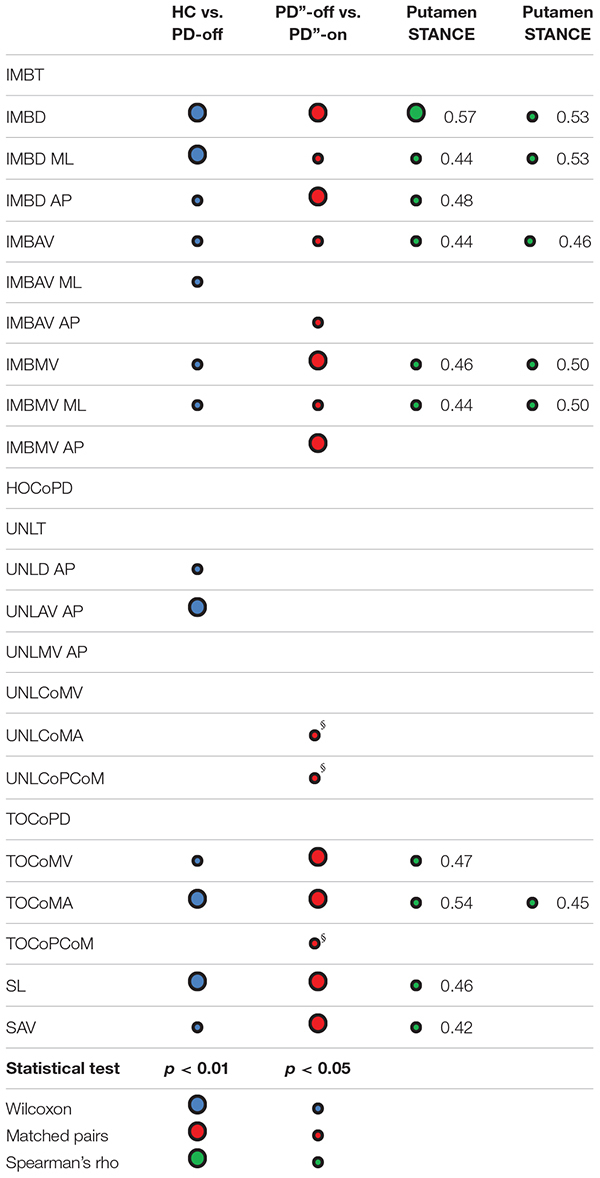

*Only significant ρ coefficients are shown. § Indicates the parameters negatively influenced (detrimental effect) by levodopa (see Table 2).*

### Levodopa Effect on Gait Initiation

All IMB measurements, and in particular the AP displacement and the average and maximum velocity (IMBD AP, IMBAV AP and IMBMV AP) significantly improved with levodopa (Table 3). The levodopa intake also positively affected the stepping phase, mostly the CoM velocity and acceleration at TO_ST_ (TOCoMV and TOCoMA) and to a lesser degree the first step length and the average velocity (SL and SAV). Of note, two measurements of the UNL phase, the AP displacement (UNLD AP) and the CoM acceleration at the end of the UNL phase [TO_SW_] (UNLCoMA) together with the distance between CoP and CoM at TO_ST_ (TOCoPCoM) worsened in the meds-on state (Table 3).

### Putaminal Dopamine Effect on Gait Initiation

With the exception of the imbalance phase AP displacement (IMBD AP), which exclusively correlated with the dopaminergic innervation of the putamen_SWING_, all other IMB measurements positively correlated with the dopaminergic innervation of both putamen (Table 3). The velocity and acceleration of CoM at TO_ST_ (TOCoMV and TOCoMA), and the first step length and average velocity (SL and SAV) correlated positively with the DAT density of the putamen_SWING_, and the TOCoMA also with the putamen_STANCE_ (Table 3).

## Discussion

The purpose of this study was to clarify the contribution of putaminal dopaminergic innervation to movement preparation in GI. We first described disease-specific biomechanical abnormalities comparing PD patients with age- and gender-matched healthy subjects; we further showed the effect of a dopaminergic therapy (i.e., levodopa) and compared kinematic resultants of APA with putaminal DAT binding values.

A preliminary but fundamental aim of this work was to investigate which measurements of the GI motor performance are influenced by AM and BoS. AM are not substantially influenced by PD (apart from particular cases such as weight change with severe dyskinesias) and their relationship with the GI parameters can be estimated and removed (O’Malley, 1996). On the contrary, individual BoS in the absence of PD is indeterminable and BoS-related parameters should be carefully interpreted and eventually excluded. Indeed, PD *per se* can variably influence the BoS according to the presence and severity of axial symptoms (e.g., rigidity, stooped posture). The variability of the effect of a chronic dopaminergic therapy on these symptoms, and the related postural compensatory adaptations, further contributes to the confounding effect of the BoS on the assessment of GI in parkinsonian patients. In this regard, only Rocchi et al. (2006) directly investigated the influence of the initial stance position on GI performance in PD. However, in this study the BoS was set with feet parallel at 5 cm or 26 cm apart, thus imposing an unnatural posture. Still, Rocchi et al. (2006) provided valuable observations on the effect of the initial stance position on GI characteristics in PD that were deepened in our study. Of most relevance, all parameters in the ML direction during the UNL phase were significantly related to the BoS (Table 2). This information is of particular value in the context of conflicting previous findings on APA production at GI in patients with PD (Burleigh-Jacobs et al., 1997; Halliday et al., 1998; Crenna et al., 2006; Jacobs et al., 2009; Schlenstedt et al., 2018). The AP displacement and velocity during the UNL phase (i.e., UNLD AP and UNLAV AP) were instead not influenced by the BoS and significantly differed between PD-off and HC (Table 2). Of note, in contrast to HC most PD patients moved the CoP backward during the UNL phase possibly to compensate for a poor CoM momentum generated during the previous IMB phase.

The assessment of the CoM and the separation of the CoP and the CoM are important features of GI, possibly determining movement performance as a result of the momentum generated with APA. Previous studies on GI from upright standing (Martin et al., 2002; Hass et al., 2005) or from a seated position (Palmisano et al., 2019) showed that CoP-CoM distance can be used to quantify poor postural stability in patients with PD. However, in our study we did not find any difference in the CoP-CoM distance in PD at the UNL end [TO_SW_] and TO_ST_. This might possibly relate to the fact that none of our patients referred any balance problem (e.g., unsteadiness, fall episodes, etc.) at the time of this study. Most relevant, is that our study showed a detrimental effect of levodopa on the CoP-CoM distance (Table 3). The values of CoP-CoM distance at unloading end (UNLCoPCoM) and CoM acceleration at unloading (UNLCoMA) above the normal range could lead to postural instability and falls, if stepping does not promptly follow the CoM perturbation. A levodopa therapy appears indeed to be a double-edged sword (Curtze et al., 2015) for dynamic balance control in PD. On one side, it favors the APA processing at basal ganglia level (IMB phase); on the other side the effect of levodopa might impair or add to compensatory adaptation thus leading to postural instability. In our study, the net effect was still favorable with an overall improvement of the stepping phase (Tables 2, 3). However, along with PD progression the disruption of APA pre-programming might cause the patients increased difficulties in mastering the GI task in meds-on state (Palmisano et al., 2019).

The main finding of our study was the correlation between the dopamine reuptake transporter (DAT) density of the putamen and almost all measurements of the CoP in the IMB phase (Table 3). Also, these measurements were significantly impaired in unmedicated patients and improved after the levodopa intake (Table 3). While a reduced AP displacement of the CoP is a consistent resultant of APA during the IMB phase at GI of PD patients (Crenna et al., 2006; Rogers et al., 2011), the correlation with the nigrostriatal dopaminergic innervation is new and provides an interesting insight into the subcortical processing of APA and feedforward motor control. The cortical-basal ganglia network appears to be bilaterally engaged during the early stages of movement preparation for the elaboration of APA, particularly of the spatial component (Table 2), for a functionally optimized GI. Increasing evidence suggests that the SMA controls the timing and planning of the APA that precede GI (MacKinnon et al., 2007; Jacobs et al., 2009), but the early buildup of preparatory cortical activity could conceivably involve the basal ganglia. It should be mentioned that the SMA is a major cortical target of the putamen (Postuma and Dagher, 2006) and dopamine modulation of SMA-putamen connectivity is fundamental for the initial encoding of movements (Coull et al., 2012). In this context, we speculate that dopamine would favor a proper selection of synergies (e.g., APA) possibly by setting an energetic-threshold for optimal motor performance depending on the current postural body schema and external disturbances. Dopamine could actually contribute in defining the optimal referent body configuration which underpins movement (e.g., gait) in the desired direction within the environment. In the context of GI, and locomotion in general, the cortico-striatal pathway would be involved in APA associated with the pre-programming of the “egocentric postural reference” during movement, which would be fundamental and preliminary for complementary circuitries (e.g., cortical-subthalamic and inter-hemispheric) to facilitate the selection and adaptation of motor programs to environmental needs (Arnulfo et al., 2018; Pozzi et al., 2019). Indeed, it has been suggested that the basal ganglia play a specific role in selecting and adapting motor programs based on internal model of body kinesthesia (Barter et al., 2015). Preliminary animal studies support this hypothesis. Barter et al. (2015) demonstrated a quantitative and continuous relationship between basal ganglia output and position coordinates during postural adaptations. Also in agreement with Barter et al. (2015), dopamine depletion could hamper the descending reference signal from the basal ganglia for orientation and configuration control in lower levels of the locomotor network (i.e., the tectum and brainstem), thus reducing the rate of change in the output (i.e., body configurations).

In conclusion our study suggests a primary role of the striatal dopaminergic circuitry in feedforward motor control during GI. Further studies will be fundamental in elucidating the contribution and interplay of other subcortical networks in APA production for the organization and execution of goal-directed voluntary movements. Great attention should be given to BoS and AM when studying GI in patients with PD.

## Data Availability Statement

The raw data supporting the conclusions of this article will be made available by the authors, without undue reservation, to any qualified researcher.

## Ethics Statement

The studies involving human participants were reviewed and approved by the University Hospital Würzburg. The patients/participants provided their written informed consent to participate in this study.

## Author Contributions

CP, GP, CF, and II contributed to study conception and design. CP, GB, and II contributed to literature research. CP, GB, NP, JB, GM, and II contributed to acquisition of data and study conduct. CP, MV, JB, AC, GM, AM, CF, and II contributed to analysis of data. CP, GB, MV, NP, JV, AM, GP, CF, and II contributed to interpretation of data. CP and II drafted and edited the manuscript. All authors contributed to the manuscript revision and gave final approval of version to be submitted.

## Conflict of Interest

The authors declare that the research was conducted in the absence of any commercial or financial relationships that could be construed as a potential conflict of interest.
